# In vitro evaluation of the inhibition potential of echinacoside on human cytochrome P450 isozymes

**DOI:** 10.1186/s12906-022-03517-0

**Published:** 2022-02-18

**Authors:** Yujie Wu, Aiqing Qiao, Shu Lin, Lijia Chen

**Affiliations:** Department of Pharmacy, The Second Affiliated Hospital of Wenzhou Medical University, No. 109, West Xueyuan Road, Wenzhou, 325027 China

**Keywords:** CYP1A2, CYP2E1, CYP3A4, CYP2C19, Herb-drug interaction

## Abstract

**Background:**

Echinacoside (ECH) possesses a wide range of biological activity. This present study analyzes the effect of ECH on cytochrome P450 isozymes (CYPs) activities of human liver microsomes.

**Methods:**

The effect of ECH on CYPs enzyme activities were studied using the enzyme-selective substrates phenacetin (1A2), chlorzoxazone (2E1), S-mephenytoin (2C19), testosterone (3A4), coumarin (2A6), diclofenac (2C9), paclitaxel (2C8), and dextromethorphan (2D6). The IC50 values for CYP1A2, CYP2E1, CYP2C19, and CYP3A4 isoforms were examined to express the strength of inhibition. Further, the inhibition of CYPs was checked for time-dependent or not, and then fitted with competitive or non-competitive inhibition models. The corresponding parameters were also obtained.

**Results:**

ECH caused inhibitions on CYP1A2, CYP2E1, CYP2C19 and CYP3A4 enzyme activities in HLMs with IC50 of 21.23, 19.15, 8.70 and 55.42 μM, respectively. The obtained results showed that the inhibition of ECH on CYP3A4 was time-dependent with the *KI/K*_*inact*_ value of 6.63/0.066 min^− 1^·μM^− 1^. Moreover, ECH inhibited the activity of CYP1A2 and CYP2E1 via non-competitive manners (*K*_*i*_ = 10.90 μM and *K*_*i*_ = 14.40 μM, respectively), while ECH attenuated the CYP2C19 activity via a competitive manner (*K*_*i*_ = 4.41 μM).

**Conclusions:**

The results of this study indicate that ECH inhibits CYP1A2, CYP2E1, CYP2C19 and CYP3A4 activities in vitro. In vivo and clinical studies are warranted to verify the relevance of these interactions.

## Introduction

Echinacoside (ECH) is a naturally occurring water-soluble and phenylethanolic glycoside compound [1]. It is widely distributed in the species of genus Cistanches and Echinacea [2, 3]. ECH is reached almost 15.5% and is a major bioactive compound in Cistanche tuhulosa (Schenk) Wight [4]. ECH possesses reported pharmacological activities such as antioxidative, neuroprotective, and antihepatotoxic activities, expected for the treatment of neurodegenerative, cardiovascular disorders, or other disorders [5–8]. Recently, studies revealed that anticancer effects of ECH have been shown in hepatocellular carcinoma [9], colorectal cancer [10], pancreatic adenocarcinoma [11], and breast cancer [12], because of its cell apoptotic-inducing function [13]. Given the numerous pharmacologically beneficial activities, ECH would often appear in combination medication clinically. Thus, the herb-drug interaction issues would subsequently arise [14]. However, it remains unresolved as to the effect of ECH on human liver microsomes cytochrome P450 isozymes (CYPs), which are mainly used to study potential drug-drug interaction (DDI).

As a series of membrane-bound hemoproteins, the CYPs were implicated in the metabolism of xenobiotics, including drugs, steroids, and carcinogens [15]. CYPs are involved in about 75% of the enzymatic reactions during the metabolism of drugs [16]. Among them, CYPs in CYP1, CYP2, and CYP3 families are the mainstay to the oxidative metabolism of 90% of clinical drugs while members in other CYPs families participate in the metabolism of endogenous compounds such as steroids, leukotrienes, and bile acids [17]. Inhibition of CYPs is a principal and major mechanism for herb-drug interactions or DDIs [18]. Drugs used alone or combined may result in DDIs mainly because of their ability to inhibit the CYPs activity [19]. According to the FDA drug-drug interaction guidance in 2020, it is routine to do in vitro assays to evaluate whether the drug involves in a metabolism-mediated DDI or which CYP will be involved in [20]. CYP1A2, CYP2B6, CYP2C8, CYP2C9, CYP2C19, CYP2D6, and CYP3A are required and CYP2E1 is one of the additional CYPs in vitro phenotyping experiments.

The present study incubated human liver CYP1A2, CYP2E1, CYP2C19, CYP3A4, CYP2A6, CYP2C8, CYP2C9, and CYP2D6 in vitro, combined with the cocktail method [21] to analyze the effect of ECH on CYPs activity of human liver microsomes (HLMs).

## Materials and methods

### Chemicals

ECH (≥98% in purity) was obtained from National Institutes for Food and Drug Control (Beijing, China). Phenacetin (≥98% in purity), chlorzoxazone (≥98% in purity), S-mephenytoin (≥98% in purity), coumarin (≥99% in purity), paclitaxel (≥95% in purity), diclofenac (≥98% in purity), furafylline (≥98% in purity), clomethiazole (≥95% in purity), tranylcypromine (97% in purity), ketoconazole (≥99% in purity), montelukast (≥98% in purity), sulfaphenazole (≥98% in purity), and quinidine (≥98% in purity) were purchased from Sigma-Aldrich (Darmstadt, Germany). Testosterone (≥97% in purity) and dextromethorphan (≥97% in purity) were from Topbiochem (Shanghai, China). Pooled (50 donors, 25 Male & 25 Female) human liver microsomes (HLMs) were purchased from PrimeTox (cat. # M10000.201800, Wuhan, China).

### Effect of ECH on human CYPs

Eight positive controls were used for different CYPs: 10 μM furafylline for CYP1A2, 50 μM clomethiazole for CYP2E1, 50 μM tranylcypromine for CYP2C19, 1 μM ketoconazole for CYP3A4, 10 μM tranylcypromine for CYP2A6, 5 μM montelukast for CYP2C8, 10 μM sulfaphenazole for CYP2C9, and 10 μM quinidine for CYP2D6. Phenotyping experiments were conducted by probe reactions of eight HLMs CYPs: CYP1A2 (by phenacetin) [22], 2E1(by chlorzoxazone) [23, 24], 2C19 (by S-mephenytoin) [25], 3A4 (by testosterone) [26], 2A6 (by coumarin), 2C8 (by paclitaxel), 2C9 (by diclofenac) [27], 2D6 (by dextromethorphan) [23] to study the effect of ECH in vitro (Table 1). As shown in Table 1, a specified amount of HLMs, substrate, and protein was pre-incubated at 37 °C with ECH for 5 min. The reaction was initiated by adding a newly prepared NADPH generating system, maintained at 37 °C for 30 min, and then terminated by the addition of acetonitrile. The incubation conditions were optimized to assure that the reaction was linear with time and protein concentration. Blank HLMs were incubated as Control. The supernatant was obtained after centrifugation at 14000 r/min, 15 min for LC-MS analysis.

**Table 1:** CYP450 isoforms tested with marker reactions at the specific incubation conditions, and *K*_*m*_ used in the inhibition study

CYPs	Reaction markers	Concentration of substrate (μM)	Concentration of protein (mg/mL)	Incubation time (min)	Estimated *K*_*m*_ (μM)
1A2	phenacetin O-deethylation	40	0.2	30	48
2E1	chlorzoxazone 6-hydroxylation	120	0.4	30	126
2C19	S-Mephenytoin 4-hydroxylation	100	0.2	40	105
3A4	testosterone 6β-hydroxylation	50	0.5	10	53
2A6	coumarin 7-hydroxylation	1.0	0.1	10	1.5
2C8	paclitaxel 6α-hydroxylation	10	0.5	30	16
2C9	diclofenac 4′-hydroxylation	10	0.3	10	13
2D6	dextromethorphan O-demethylation	25	0.25	20	4.8

### LC–MS/MS analysis

The quantification for the corresponding metabolites was performed using a validated single LC/MS/MS run at an Ultimate 3000 HPLC system and a TSQ Quantis™ triple quadrupole mass spectrometer equipped with a heated electrospray ionization (ESI) source (Thermo Fisher, USA) [28]. The mobile phase was consisted of solvent A (0.01% acetic acid and 5 mmol/L ammonium acetate) and solvent B (methanol-acetonitrile,1:1, v/v) according to the following gradient: 0-0.5 min, B 2%; 0.5-4.0 min, B 2 to 45%; 4.0-6.5 min, B 45 to 60%; 6.5-6.8 min, B 60 to 80%; 6.8-7.2 min, B 80%; 7.2-7.5 min, B 80 to 2%; 7.5-10.0 min, B 2%. The separation of metabolites was achieved in a Phenomenex Luna C18 100A column (2.0 mm × 150 mm, i.d. 5 mm) at a flow rate of 0.5 mL/min in a column oven at 40 °C. The parameters of interface ESI were as follows: nebulizing gas flow, 3 L/min; heating gas flow, 15 L/min; interface temperature, 350 °C; DL temperature, 250 °C; heat block temperature, 400 °C; drying gas flow, 5 L/min. This LC-MS/MS method was validated in advance with a matrix effect ranged from 67 to 134% and the recoveries from 96 to 108%. All of the analytes fulfilled the LLOQ criterion of signal-tonoise ratio of 10:1.

### Enzyme inhibition studies of ECH on CYP1A2, CYP2E1, CYP2C19 and CYP3A4

According to the result of ECH inhibitory effect, the CYPs, whose activities were strongly inhibited, were subjected to half-maximal inhibitory concentration (IC50) assay. Briefly, reaction mixtures containing HLMs, substrate and protein were incubated with a series of ECH solutions with different concentrations (0, 2.5, 5, 10, 25, 50, 100 μM). The relative enzyme activity was observed by High-Performance Liquid Chromatography (HPLC)-Mass Spectrometry (MS). *IC*_*50*_ values were calculated by Graphpad Prism 7.

### Assessment of time-dependent inhibition

To clarify the inhibition mechanism of ECH on CYP1A2, CYP2E1, CYP2C19 and CYP3A4, the activity of these four CYPs were detected at different times at the presence of 20 μM, 20 μM, 10 μM or 50 μM ECH respectively. In brief, ECH was pre-incubated with HLMs generated by NADPH system for 30 min at 37 °C. After the first incubation, an aliquot (20 μL) was transferred to another incubation tube, added probe substrates and start the reaction by an NADPH-generating system. After incubation for 0, 5, 10, 15, and 30 min, the corresponding metabolites were determined by HPLC-MS.

### Effects of ECH on the kinetic parameters of CYP1A2, CYP2E1, CYP2C19 and CYP3A4

To evaluate the kinetic mechanisms of ECH towards CYP3A4, the relative remaining activities of CYP3A4 were determined at incubation time with or without ECH. Microsomal reaction mixtures were pre-incubated for 0, 5, 10, 15, 30 min with or without ECH at 0, 2, 5, 10, 20, 50 μM.

To evaluate the kinetic mechanisms of ECH towards CYP1A2, CYP2E1, and CYP2C19, the incubations were conducted using different substrate concentrations and various ECH concentrations with a two-step incubation scheme, and the relative remaining activities were determined.

### Statistical analysis

Graphpad Prism 7 was used for all statistical analyses. All experiments were performed at least thrice. Comparisons were analyzed between two groups using two-sided Student’s t-test and among multiple groups using one-way analysis of variance (ANOVA). The prediction of the IC50 values was done by plotting relative CYP activities vs. the logarithm of ECH concentrations.

The kinetics of the index reactions by CYPs were described by one of the following models:

CYPs time-dependent inhibition:

$${K}_{obs}=\frac{K_{inact}\left[I\right]}{K_I+\left[I\right]}$$

A one enzyme Michaelis Menten model with competitive substrate inhibition:

$$V=\frac{{\mathrm{V}}_{max}\mathrm{S}}{{\mathrm{K}}_m\left(1+\frac{I}{K_I}\right)+\mathrm{S}}\mathrm{a}$$

Michaelis-Menten model with uncompetitive substrate inhibition:

$$V=\frac{{\mathrm{V}}_{max}\mathrm{S}}{{\mathrm{K}}_m+\mathrm{S}\left(1+\frac{I}{K_I{K}_i}\right)}$$

*I*: the concentration of the ECH; *K*_*I*_: the inhibition constant; *K*_*inact*_: inactivation rate constant; S: the concentration of the substrate: *K*_*m*_:

The inhibitory effects of ECH on CYP enzymes are presented graphically as Lineweaver-Burk’s plots indicating *K*_*i*_ values.

## Results

### ECH inhibits the activity of CYP1A2, CYP2E1, CYP2C19 and CYP3A4

**Fig. 1:**
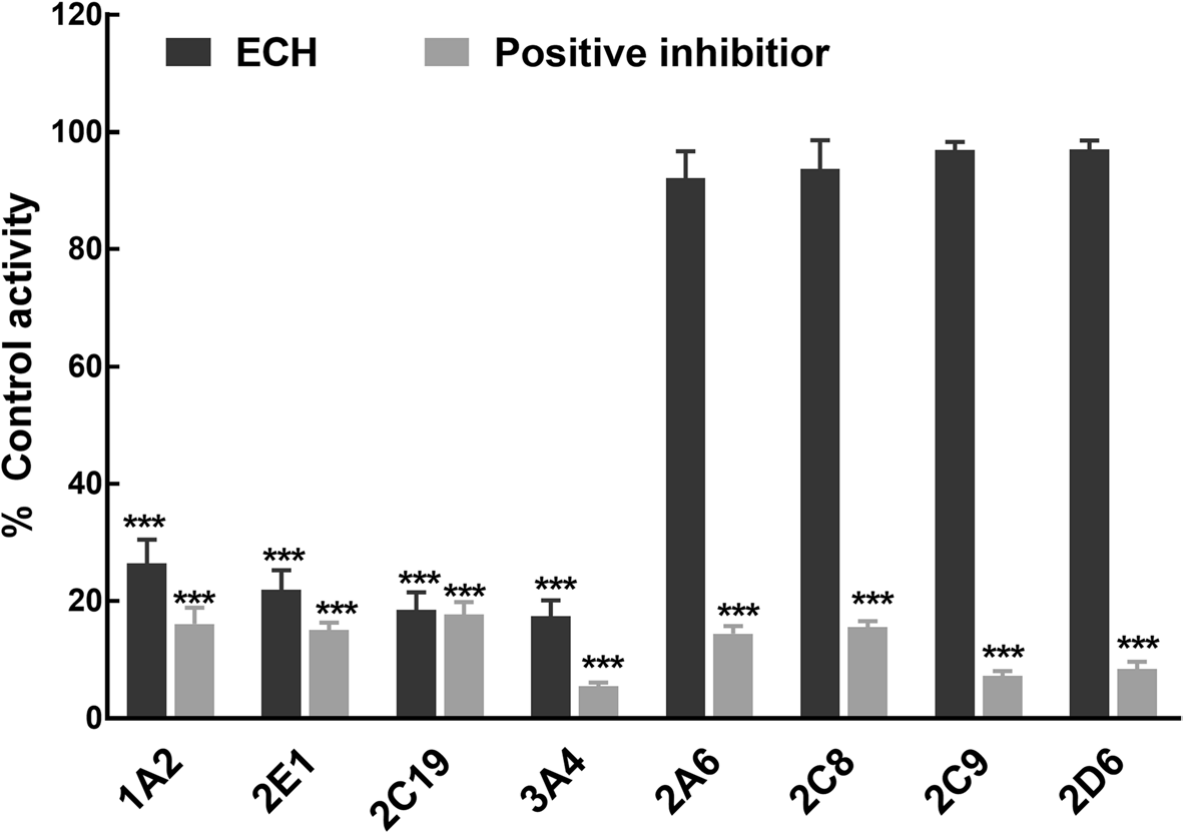
Effects of ECH on metabolite formation are shown as indexes of CYPs activity in HLM (CYP1A2, CYP2E1, CYP2C19, CYP3A4, CYP2A6, CYP2C8, CYP2C9, and CYP2D6). ECH: incubation with 100 μM ECH. Positive inhibitor: incubation with specific inhibitors, phenacetin (for CYP1A2), chlorzoxazone (for CYP2E1), S-mephenytoin (for CYP2C19), testosterone (for CYP3A4), coumarin (for CYP2A6), paclitaxel (for CYP2C8), diclofenac (for CYP2C9), dextromethorphan (for CYP2D6). Blank HLMs was incubated as Control. ^***^P < 0.001 vs Control activity. ECH: echinacoside

**Fig. 2:**
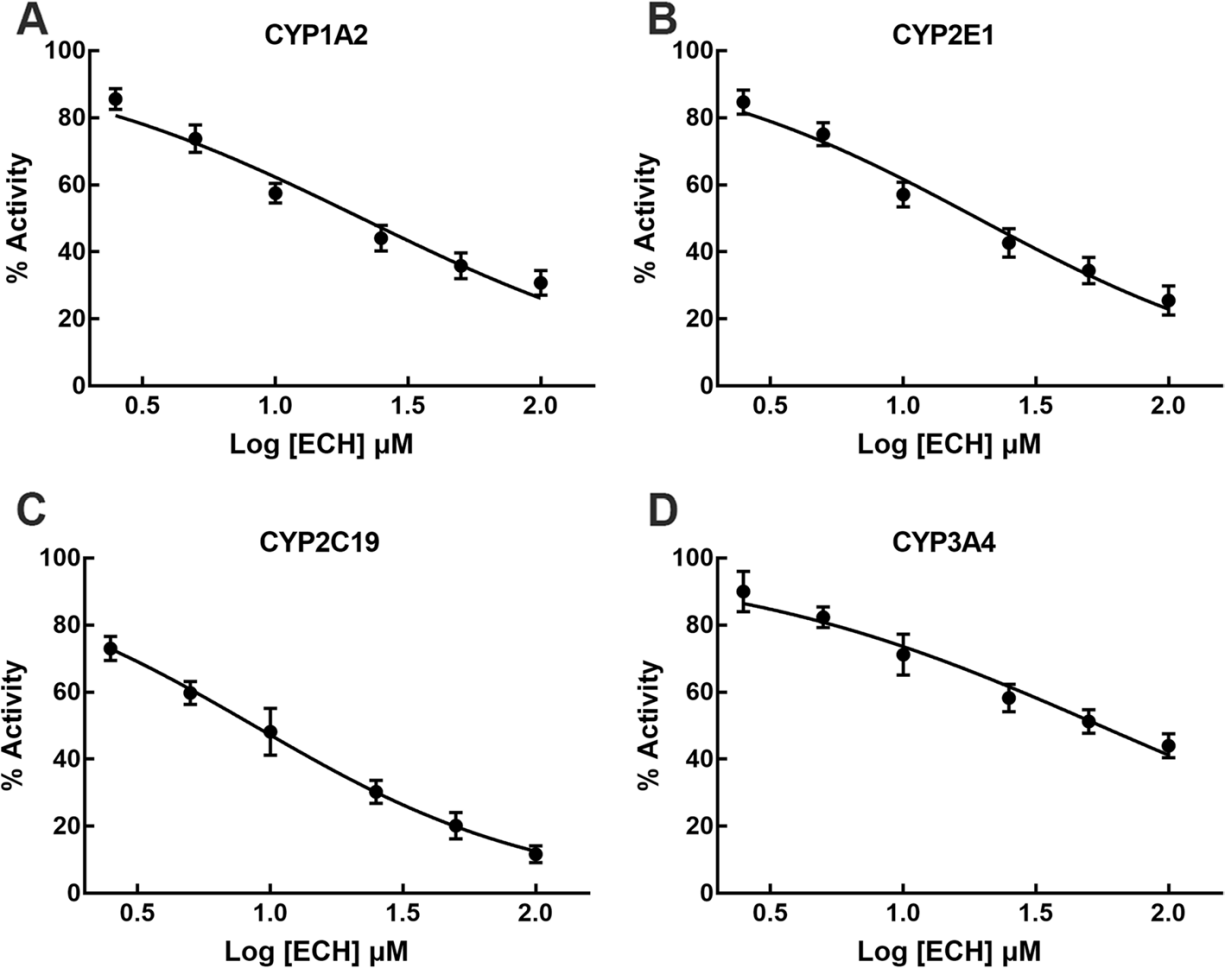
The inhibitory effect of ECH on four main CYP isoform activities in pooled HLMs: (**A**) CYP1A2; (**B**) CYP2E1; (**C**) CYP2C19; (**D**) CYP3A4

To investigate whether ECH affects the catalytic activity of CYPs, the probe reaction assays were conducted with various concentrations of ECH in HLM. It is found the presence of ECH caused a significant decrease in the relative activity of HLMs CYP1A2, CYP2E1, CYP2C19 and CYP3A4 (*p* < 0.001, Fig. 1). ECH leads to an inhibition of CYP1A2, CYP2E1, CYP2C19 and CYP3A4 enzyme activity in HLMs with *IC*_*50*_ of 21.23 (95%CI: 18.27 to 24.79), 19.15 (95%CI: 16.84 to 21.84), 8.70 (95%CI: 7.81 to 9.67) and 55.42 Μm (95%CI: 45.8 to 69.27), respectively (Fig. 2). However, ECH showed no significant inhibition of CYP2A6, CYP2C8, CYP2C9 and CYP2D6 in HLM even in 100 μM concentrations.

### The inhibition of CYP3A4 by ECH was time-dependent

**Fig. 3:**
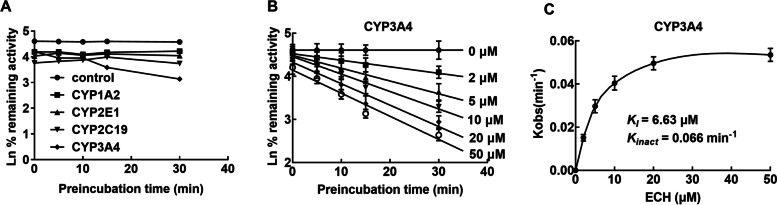
Time-dependent inhibition of ECH on CYP3A4. Microsomal reaction mixtures were pre-incubated for 0, 5, 10, 15, 30 min with or without ECH at 0, 2, 5, 10, 20, 50 μM. **A** The inhibitions of ECH on CYP1A2, CYP2E1, and CYP2C19 were time-independent, while the inhibition of ECH on CYP3A4 was time-dependent. **B** ECH inhibited CYP3A4 in a time-dependent manner. **C** The value of Kobs is plotted against the concentration of the inactivator to determine *KI* and *K*_*inact*_. ECH: echinacoside

To explore the inhibition of ECH on CYPs was time-dependent or not, the activities of CYP1A2, CYP2E1, CYP2C19 and CYP3A4 at different times with or without ECH were detected. CYP3A4 was found to decrease with the prolongation of incubation time with ECH which suggests that ECH may be a time-dependent inhibitor of CYP3A4, while activities of CYP1A2, CYP2E1 and CYP2C19 can’t be influenced by the incubation time (Fig. 3A). As a result, time-dependent inhibition of the CYP3A4 isoform was further evaluated, and the *K*_*I*_ and *K*_*inact*_ could be estimated from the curve fitting as 6.63 μM and 0.066 min^− 1^, respectively^− 1^ (Fig. 3 B and C).

### Noncompetitive inhibition of ECH on CYP1A2 and CYP2E1

**Fig. 4:**
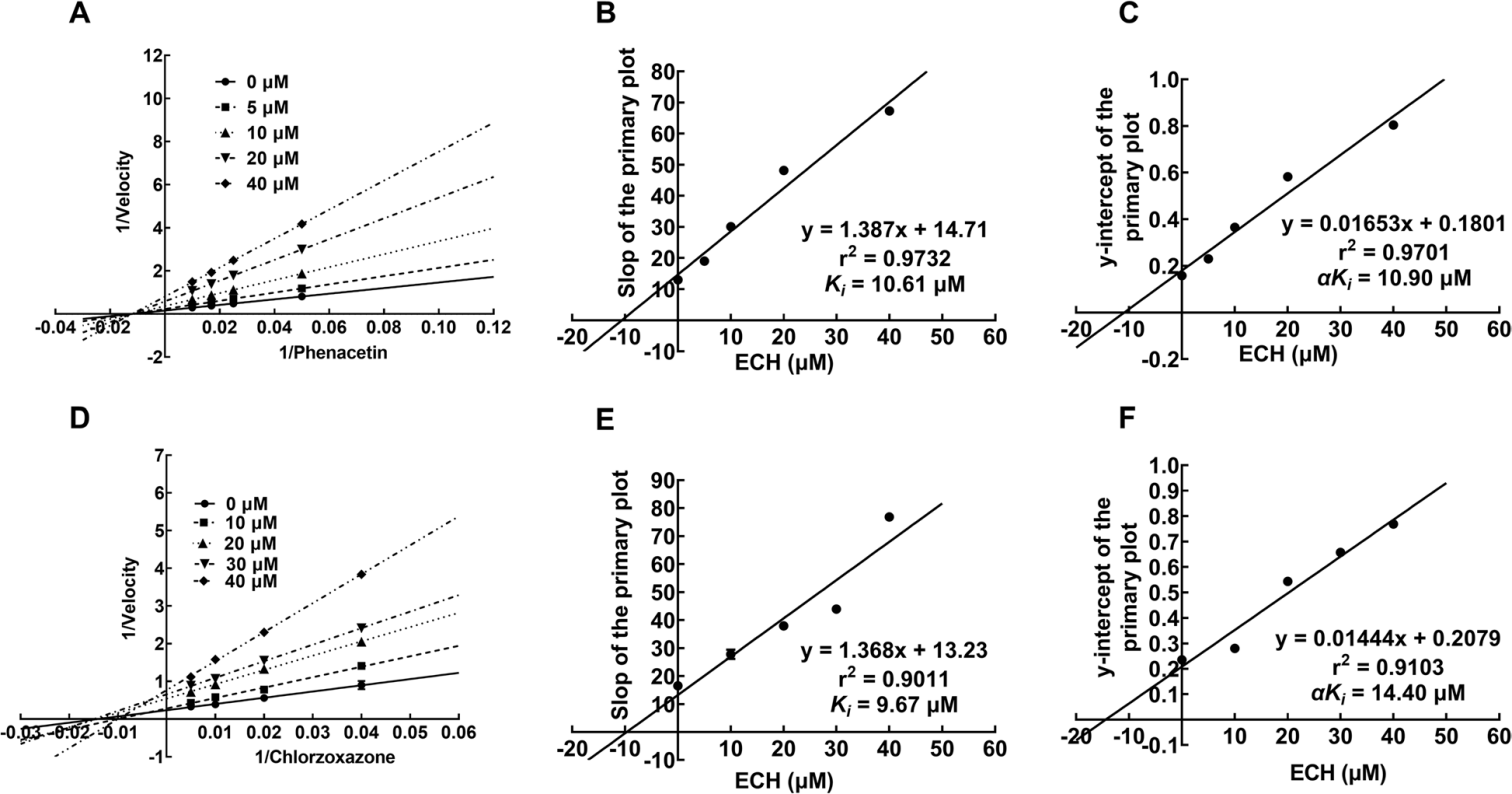
Primary Lineweaver-Burk plots, the secondary plot for *Ki*, and the secondary plot for α*Ki* of CYP1A2 (**A**-**C**) and CYP2E1 (**D**-**F**) inhibited by ECH

Given the inhibition of CYP1A2 and CYP2E1 by ECH was not time-dependent, we further their inhibitory mechanism in vitro. Hence the mode of inhibition and *Ki* values of ECH was determined for CYP1A2, CYP2E1 enzymes in HLMs. Lineweaver-Burk plots of ECH for CYP1A2 and CYP2E1 inhibition in HLMs are shown in Fig. 4. Given the α*Ki* was 10.90 μM for CYP1A2, and the α value was approximately 1, the inhibition of ECH on CYP1A2 was estimated as noncompetitive inhibition (Fig. 4 A-C). The αKi was 14.40 μM for CYP2E1, and the α value was not 1, which suggests the inhibition of ECH on CYP2E1 was mixed type (Fig. 4 D-F).

### Competitive inhibition of ECH on CYP2C19

**Fig. 5:**
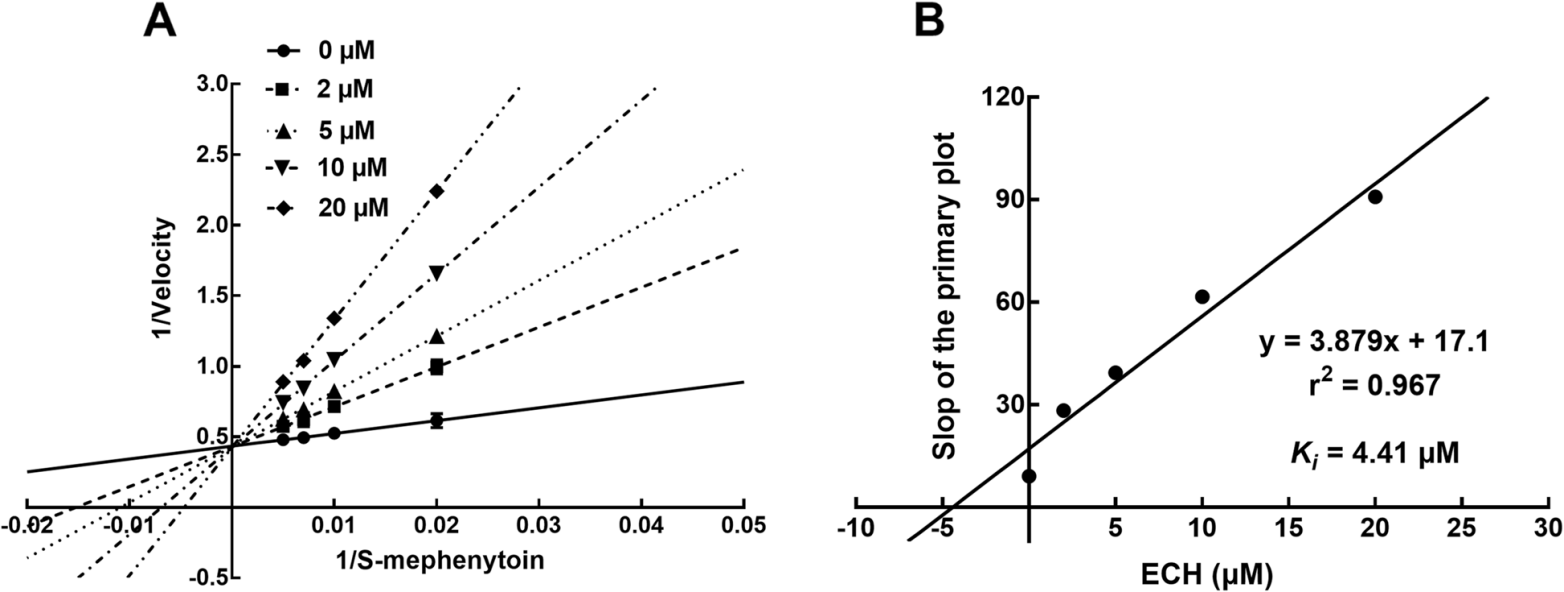
Primary Lineweaver-Burk plots (**A**) and the secondary plot for *Ki* (**B**) for α*Ki* of CYP2C19 inhibited by ECH

By the Lineweaver-Burk plots, the inhibition of CYP2C19 by ECH showed as competitive type with the *Ki* value of 4.41 μM, (Fig. 5).

## Discussion

CYPs inhibition is known as the main mechanism for metabolism-based drug-drug interactions [29]. Some drugs compete for the same enzyme binding sites. When these drugs were taken simultaneously, the interaction usually happen [30]. It is well documented that CYPs inhibition impairs the clearance or biotransformation of drugs resulting in elevated plasma levels that influence therapeutic efficacy or even increase the probability of adverse drug reactions [31]. As a potent drug with a diversity of biological activities and given the fact that ECH is enriched in commonly used herbs, so we intend to explore its effect on human CYPs to provide the basic mechanism of ECH-drug interaction. Based on the mechanism, inhibition of CYPs can be classified into reversible and irreversible (competitive or noncompetitive) inhibition [30].

As recommended by FDA guidelines for elucidation of potential drug interactions during the drug development process, eight HLMs CYPs (CYP1A2, CYP2B6, CYP2C8, CYP2C9, CYP2C19, CYP2D6, CYP3A4, and CYP2E1) were used in this study. The investigational ECH was initially evaluated in this study for potential interactions with CYPs in vitro. Preliminary findings from in vitro experiments in HLMs demonstrated that ECH was an inhibitor of multiple CYP enzymes, including CYP3A4, CYP1A2, CYP2E1, and CYP2C19. With *IC*_*50*_ values of 21.23 and 19.15 μM, ECH was a moderate inhibitor for CYP1A2 and CYP2E1. With an *IC*_*50*_ value of 8.70 μM, ECH was a strong inhibitor for CYP2C19. For CYP3A4, though the *IC*_*50*_ value of 55.42 μM, ECH caused time-dependent inhibition of CYP3A4 in HLMs in vitro. In addition, ECH inhibited CYP1A2 in a non-competitive manner, competitively inhibited CYP2C19, and inhibited CYP2E1 in a mixed manner.

CYP1A2 is one of the major CYPs in the human liver, accounts for about 13% of human liver CYPs. CYP1A2 is notable for its capacity to N-oxidize arylamines and is responsible for the metabolism of a broad range of arylamines and heterocyclic arylamines in therapeutic drugs such as phenacetin, lidocaine, and tacrine [32]. CYP1A2 and other members in the CYP1A family are responsible for the metabolism of more than 9% of marketed drugs, especially caffeine, theophylline, and melatonin. Given CYP1A2 may undergo inhibition or induction by substrate drugs or other widely used agents, drug interactions mediated by CYP1A2 are a key issue in clinical practice. However, only a small proportion of the potential interactions have been studied so far. Therefore, the prediction of CYP1A2-mediated drug interactions is considerably desirable. In this study, we measure the inhibition of ECH on CYP1A2, and found ECH had a moderate non-competitive inhibition on human CYP1A2 in vitro. Co-administration of sensitive CYP1A2 substrate drugs with their inhibitors may lead to a large increase in substrate drug exposure and drug interaction. For instance, the substrates such as agomelatine, melatonin or tizanidine would be with 5-fold exposure if they are co-administered with CYP1A2 inhibitors such as fluvoxamine, rofecoxib or ciprofloxacin [33]. This study provides basic research into the potential drug-drug interaction of ECH related to CYP1A2.

CYP2E1 is identified as a significant contributor to drug-induced hepatotoxicity since it involves the bioactivation of more than 85 xenobiotics [34]. CYP2E1 was recently characterized to be the highest expressed CYP enzyme in human livers. CYP2E1-related toxicity is associated with its protein level that shows significant inter-individual variability. CYP2E1 contributes to the metabolism of some widely used drugs, such as acetaminophen and chlorzoxazone, and is associated with nephrotoxicity and hepatotoxicity of cisplatin [34]. In addition, CYP2E1 mediated biotransformation plays a vital role in the formation of macromolecular adducts, which can cause organ damage such as liver cirrhosis. Thus, inhibiting CYP2E1 has potential significance in reducing the toxicity of macromolecular xenobiotics [35]. In the present study, ECH showed moderate inhibition of CYP2E1, which shed light on the development of a potential CYP2E1 inhibitor.

CYP2C19, though a minor hepatic CYP, metabolizes up to 15% of known clinically pertinent drugs, including drugs with narrow therapeutic windows but frequently encountered by physicians such as warfarin, carbamazepine, and clopidogrel [36]. ECH strongly inhibited the enzyme activities of CYP2C19, in a concentration-dependent manner in our study. This would be a warning sign for the possibility of drug interactions between ECH and drugs with narrow therapeutic windows.

CYP3A4 plays a prominent role in drug metabolism. Approximately 65% of drugs approved by the US Food and Drug Administration were CYP3A4 substrates, 30% were inhibitors, and another 5% were inducers [37]. Therefore, CYP3A4 is a major locus for problems with drug-drug interactions [38]. Our results suggested that ECH as a time-dependent inhibitor for CYP3A4 though showed weak inhibition. Given that time-dependent inhibition is considered as an irreversible or quasi- irreversible type of enzyme inhibition, ECH could have a persistent effect on CYP3A4 activity in the human liver because only de novo synthesis of CYP3A4 would overcome the time-dependent inhibition after the intake of ECH stop. This could lead to further risks for drug-drug interactions. This study provides knowledge about the inhibitory potential of ECH on CYP3A4-mediated metabolism in vitro that would benefit for minimizing the possibility of drug-drug interactions when co-administered with other drugs in vivo.

Previously, echinacea preparations have been reported to be responsible for inhibiting CYP3A4, CYP1A2 and CYP2C9 [5, 39]. In vivo, the co-administration of echinacea and theophylline (with a narrow therapeutic window) or clozapine, tacrine, and cyclobenzaprine (having adverse effect concerns) may give rise to an increased incidence of toxicity or adverse reaction [40]. This is consistent with the inhibition of hepatic CYP1A2, CYP2E1, CYP2C19, and CYP3A4 by ECH in our study. However, ECH, as a caffeic acid conjugate, could not be identified in human plasma samples after echinacea tablet ingestion [5]. Notably, the strength of the interaction varies a lot among different genetic polymorphism of CYPs, such as CYP2D6 and CYP2C19 [41]. Further in vivo studies need to be investigated to determine the distribution of ECH in vivo and verify the potential of ECH on each CYP genetic polymorphism, to establish appropriate recommended dose for the safe therapeutic use of ECH or related herbal medicines.

## Conclusion

In conclusion, ECH inhibited CYP1A2-, CYP2E1-, CYP2C19- and CYP3A4- mediated metabolism at different degrees. ECH moderately inhibited the activity of CYP2C19, slightly inhibited the activities of CYP1A2 and CYP2E1, and inhibited the activity of CYP3A4 in a time-dependent manner. So, it requires careful attention when ECH or herbal medicines containing ECH co-administrated with other drugs, which sharing the same CYPs, especially those with a narrow therapeutic window or having adverse effect concerns.

## Data Availability

The corresponding author may provide data and materials related to this study.
